# The Effect of Simulated Leg-Length Discrepancy on the Dynamic Parameters of the Feet during Gait—Cross-Sectional Research

**DOI:** 10.3390/healthcare9080932

**Published:** 2021-07-24

**Authors:** Héctor Pereiro-Buceta, Ricardo Becerro-de-Bengoa-Vallejo, Marta Elena Losa-Iglesias, Daniel López-López, Emmanuel Navarro-Flores, Eva María Martínez-Jiménez, João Martiniano, César Calvo-Lobo

**Affiliations:** 1 Research, Health and Podiatry Group, Department of Health Sciences, Faculty of Nursing and Podiatry, Universidade da Coruña, 15403 Ferrol, Spain; hector.pereiro@udc.es (H.P.-B.); daniellopez@udc.es (D.L.-L.); 2 Faculty of Nursing, Physiotherapy and Podiatry, Universidad Complutense de Madrid, 28040 Madrid, Spain; ribebeva@ucm.es (R.B.-d.-B.-V.); evamam03@ucm.es (E.M.M.-J.); cescalvo@ucm.es (C.C.-L.); 3 Faculty of Health Sciences, Universidad Rey Juan Carlos, 28922 Alcorcón, Spain; marta.losa@urjc.es; 4 Frailty Research Organizaded Group (FROG), Department of Nursing, Faculty of Nursing and Podiatry, University of Valencia, 46010 Valencia, Spain; 5 Escola Superior de Saúde da Cruz Vermelha Portuguesa de Lisboa, 1300-125 Lisbon, Portugal; jmartiniano@esscvp.eu

**Keywords:** leg length inequality, gait, reliability analysis, pressure platform, lower limbs

## Abstract

Background: The effect of Leg-Length Discrepancy (LLD) on dynamic gait parameters has been extensively discussed. Podobarography is the study of foot-to-ground pressure distribution. It has been used to test plantar footprint deviations that could reveal pathology. Purpose: The aim of this study is to determine the effects of simulated LLD on dynamic gait parameters measured with a pressure platform in healthy subjects. Methods: Thirty-seven healthy subjects participated in observational cross-sectional research. A procedure was performed to capture the dynamic parameters of each participant under five different simulated LLD conditions. Support time, mean pressure, and peak pressure measures were registered on three trials for each foot and LLD level per session. An analysis of variance (ANOVA) test for repeated measures was performed to check for differences between the different simulated LLD levels. Results: The stance time of the short leg had no significant changes. The stance time of the long leg increased by 3.51% (*p* < 0.001), mean pressure of the short leg increased by 1.23% (*p* = 0.005), and decreased by 5.89% in the long leg (*p* < 0.001). Peak pressure of the short leg decreased by 2.58% (*p* = 0.031) and the long leg decreased by 12.11% (*p* < 0.001). Conclusions: This study demonstrates that increasing LLD causes an asymmetrical foot-loading pattern, with decreased mean and peak pressure on the longer limb, and consequently an overload on the short side. Furthermore, an increasing LLD causes increased stance time on the long leg.

## 1. Introduction

Leg-length discrepancy (LLD) is a condition frequently described in the literature as the unequal length of lower limbs. It is estimated to involve 40–70% of the population and can exceed 2 cm of inequality in close to 0.1% [1]. Knutson et al. concluded in a meta-analysis of 573 subjects that only 10% of the population had equal-length lower limbs [2]. LLD has been a cause of controversy in the clinical and research community for a long time. There is no agreement on many facets, such as its impact on various neuromusculoskeletal disorders, assessment of measurement methods, prevalence, and the degree of its clinical significance [3].

LLD can be classified as anatomical when the difference is caused by structural deformities that can be measured directly in the lower limb bones, or as functional when the inequality is caused by postural defects [4]. Both categories have been associated with hip or knee osteoarthritis and other mechanical pathologies as a result of an incorrect distribution of load [5,6].

Asymmetries in the kinematics of gait have been associated with different degrees of true LLD [5]—essentially pelvic drop and hip adduction in the stance phase [7,8]. Several authors have found flexion anomalies in the sagittal plane of the hip, knee, and ankle [7,9]. Furthermore, LLD has been related to decreased load times, stride length, and gait velocity of the shorter limb and increased cadence [10]. Kinetics asymmetries induced by LLD also appear to be related to the etiology of plantar fasciitis, lower back pain, and knee [11,12,13].

Clinically, two methods are commonly used to measure LLD: the direct method, which measures the distance between anterior superior iliac spine and medial malleolus when in a supine decubitus using a calibrated tape measure [13]; and the indirect method, measuring LLD using lifts to level the pelvis, rather using a pelvic leveling device, in a standing position [14]. Radiographic scanogrammetry is considered the gold standard for limb-length measurement but exposes patients to ionizing radiation [15]. These methods cannot assess dynamic changes in leg length, as they are performed in a static position.

In the literature, there are two ways to approach the study of LLD: examining subjects with diagnosed LLD, or simulating LLD on healthy subjects, appraising its role on gait anomalies. Some authors consider the first method to be limited by physical anomalies that commonly develop in subjects with real LLD as a result of compensations. Thus, these cannot be treated as pure LLD subjects [3].

Betsch et al. described a non-invasive method to simulate and evaluate LLD and its impact on human gait using plantar lifts [16,17].

Podobarography is the study of foot-to-ground pressure distribution. It has been used to evaluate foot static and dynamic interactions with terrain, posture [18], and the screening of plantar footprint deviations that could reveal pathology [19].

The aim of this study is to analyze the effect of simulated LLD on dynamic parameters obtained from a pressure platform in normal individuals. We hypothesized that subjects would asymmetrically alter their foot-loading pattern.

## 2. Materials and Methods

### 2.1. Design and Sample

The sample size was calculated with software from Grupo de Investigación en Riesgo Cardiovascular y Nutrición and Grupo de Investigación en Epidemiología y Genética Cardiovascular, IMIM-Hospital del Mar. Barcelona [20], to detect the correlation between dynamic parameters of the gait and different degrees of simulated LLD, which were standard deviation (SD) 4.34–3.48 [21] with 80% statistical power (β = 20%) and an interval of confidence 95% (α = 0.05) and 2-tailed test. A total of 37 participants were required to detect a difference equal or higher than 0.4 units. An SD of 0.86 and loss to follow-up rate of 0% is assumed.

Thirty-seven healthy test subjects (13 men and 24 women) aged 19 to 61 years old participated in the study. An observational cross-sectional research design according to Strengthening The Reporting of Observational Studies in Epidemiology (STROBE) criteria [22] and non-random consecutive sampling technique were used. The inclusion criteria were: being over 18 years, a European footwear size of 36–45, and no history of musculoskeletal damage or pain during the last year. Furthermore, clinical exploration was accomplished by the principal researcher to exclude real LLD > 5 mm, limited joint range, or asymmetrical pronated feet [23].

### 2.2. Ethical Considerations

The Research and Ethics Committee of Universidad Rey Juan Carlos, Spain, issued a favorable authorization certificate n° 0904201907519 for this study, following the ethical principles of the Helsinki declaration [24]. All subjects signed an informed consent before participating in this study.

### 2.3. Dynamic Data Collection

LLD was simulated with Ethyl-Vinyl-Acetate plantar lifts of 70A shore hardness and 5, 10, 15, and 20mm height, secured to the right shoe of each subject. This proceeding emulates LLD by generating pelvic obliquity.

In order to capture the dynamic parameters, we used a Podoprint^®^ platform (Namrol Group, Barcelona, Spain), which, in a previous publication, was used to assess the intra and intersession repeatability and reliability in healthy subjects with simulated LLD [25]. A self-calibrating system, with 1600 10 × 10 mm resistive sensors and a sample rate of 100 Hz, was installed into the center of a flat 4.8 m walkway at ground level (Table 1).

Each volunteer was instructed to walk normally, looking straight ahead. The starting position was set to match the footstep on the platform. Participants walked at a self-selected speed for all the trials; however, it was controlled with digital video recording to ensure the normal cadence under laboratory conditions, which range from 81 to 138 steps per minute [26]. The procedure was performed to register the dynamic parameters of each participant under five different simulated LLD levels (0, 5, 10, 15, and 20 mm) in randomized order. Stance time (ms), mean pressure (g/cm^2^), and peak pressure (g/cm^2^) measures were recorded. These parameters were considered the most frequently employed in functional foot assessment of pathological conditions [27,28,29].

**Figure 1:**
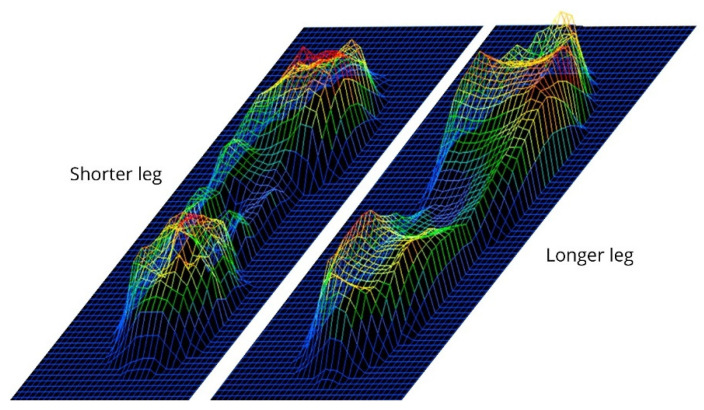
An example of the plantar pressure curves of one subject during gait with a simulated LLD (20 mm).

Two testing sessions were held on seven separate days. Three trials were performed for each foot and LLD level per session. Before capturing the dynamic data, all volunteers completed a three-minute walk on the walkway to habituate themselves with the platform and plantar lifts. Four steps of each foot were collected per trial using the platform’s “Multiple Dynamic” mode, which directly provides the averaged parameters. The sample rate was 100 Hz. The same researcher tested all the participants (Figure 1). The data obtained from the pressure platform system were stored and processed using manufacturer-specific software Podoprint^®^ for Windows^®^, version 8.6.5 (Namrol Group, Barcelona, Spain).

### 2.4. Statistical Analysis

All data were verified for outliers and normal distribution by the one-sample Shapiro–Wilk test. Normally distributed data were presented as mean and standard deviation. Samples were removed individually if found to be greater than 3 SD from the group mean. The intrasession reliability was obtained by three repeated trials for each simulated DLL condition and each foot at the first and second testing sessions. The coefficient of variation (CoV) [30] was used to indicate the relationship between the size of the mean and the variability of each of the variables studied and it was calculated as CoV(%)=DS/Mean*100. The intraclass correlation coefficient (ICC) obtained using the (2,1) model (two-way random, single measurement, absolute agreement ICC model) was calculated in order to analyze the reliability between trials [31]. The standard error of measurement (SEM) was calculated as $SEM=SD\sqrt{\left(1-ICC\right)}$ and expressed as a percentage of the mean: SEM%=SEM/Mean*100 [32].

Furthermore, the minimum detectable change (MDC) was calculated. This was described as the magnitude of the value variation of each scale, below which change can be interpreted as inherent to the variability of the measurement method, without any real change to the clinical situation of the subject. It was obtained as a standardized mean as $MDC=1.96*SEM\sqrt{2}$ and expressed as a percentage of the mean: MDC%=MDC/Mean*100 [33,34].

Intersession reliability was determined by retesting all subjects seven days after the first session. The average of the measurements for each session, for each subject and LLD condition, was used to calculate the ICC^3,1^. For absolute comparison of the results obtained in the two sessions, CoV, SEM and MDC were expressed as percentages of the mean [33,34].

An analysis of variance (ANOVA) test for repeated measures was performed to check for differences between the different simulated LLD levels [35]. Bonferroni was used to adjust the type I error for multiple comparisons. To verify sphericity assumption, Mauchly’s test was used and subsequently corrected for lack of sphericity using the Greenhouse–Geisser correction. The level of significance was set at *p* < 0.05. To estimate the effect size, partial eta squared (η^2^_p_) were calculated. Cohen [36] provided benchmarks to define small (η^2^ = 0.01), medium (η^2^ = 0.06), and large (η^2^ = 0.14) effects.

The IBM^®^ SPSS^®^ for Windows^®^, version 22.0 statistical package, was used for data analysis and graphics (SPSS, Inc., Chicago, IL, USA).

## 3. Results

Thirty-seven healthy test subjects (13 men and 24 women) aged 19 to 61 years participated in the study (Table 2).

The measurements were reproducible for an individual even if repeated during the same test session or when tested seven days later. Descriptive statistics, represented by mean and standard deviation, and reliability data, represented by CoV, ICC, SEM and MDC, were calculated for the first session. The Cov for intrasession reliability ranged from 0.13% to 2.13%, and the ICC ranged from 0.760 to 0.980. The SEM% ranged from 0.03% to 0.70%, and the MDC% ranged from 0.07% to 1.93%.

In the second session, CoV for intrasession reliability ranged from 0.12% to 1.64%, and the ICC ranged from 0.771 to 0.980. The SEM% ranged from 0.03% to 0.78%, and MDC% ranged from 0.08% to 1.91%.

The average measurements from both test sessions and intersession reliability data, represented by CoV, ICC, SEM% and MDC%, are presented in Table 3. The CoV for intersession reliability ranged from 0.39% to 1.65%, and the ICC ranged from 0.866 to 0.988. The SEM% ranged from 0.06% to 0.58% and MDC% ranged from 0.18% to 1.61%.

The results of repeated-measures ANOVA showed statistically significant changes in five of six studied variables under different simulated LLD conditions (Table 4). In addition, a pairwise comparison based on the estimated marginal means between different LLD levels of each dynamic variable was calculated (Table 5).

**Figure 2:**
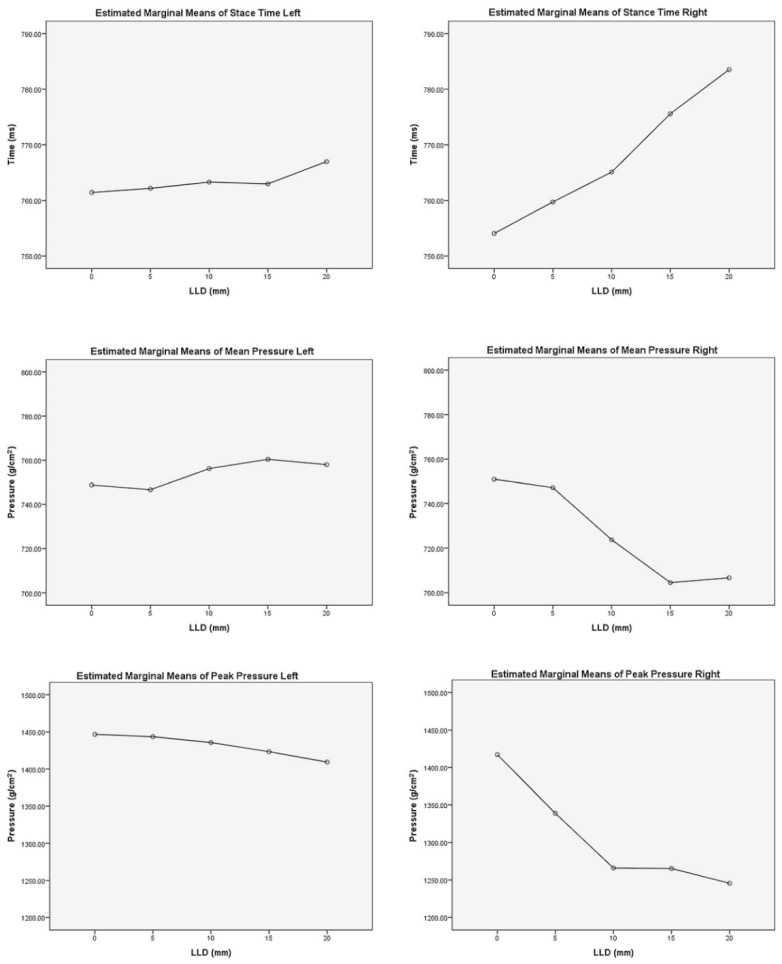
Estimated marginal means of stance time, mean pressure and peak pressure.

Changes were observed in all pressure parameters, in the short and long leg, and in the stance time of the long leg: stance time of the long leg increased by 3.51% (*p* < 0.001), mean pressure of the short leg increased by 1.23% (*p* = 0.005) and decreased by 5.89% in the long leg (*p* < 0.001). Peak pressure of the short leg decreased by 2.58% (*p* = 0.031) and decreased by 12.11% in the long leg (*p* < 0.001). (Figure 2)

## 4. Discussion

Different reports expose that LLD can generate substantial gait parameters deviations [3]. We can find in the literature two methods to evaluate LLD effects on gait: measuring subjects with real LLD or simulating LLD on healthy subjects. Using subjects with real LLD, it is more difficult to obtain a homogeneous sample, due to a greater diversity of LLD, ages, or associated pathologies that could potentially lead to confounding variables.

The objective of the current study was to understand the effect of simulated LLD on dynamic parameters measured with a pressure platform in healthy subjects. The number of participants was 37, a total of 74 limbs were evaluated, comprising 444 measurements.

This study demonstrates that as LLD increases, there is a decreased mean pressure and peak pressure on the long leg. As a result, the subjects asymmetrically alter their foot loading pattern. According to White [37], the shorter limb for the simulated LLD group supported greater loads and loading rates. In his study, the results showed short-term responses to an induced change in LLD. Over time, asymmetric limb loading could be reduced by compensatory strategies [38]. Disproportionate pressures and higher load rates under dynamic conditions could make the shorter limb susceptible to limb joint damage. Golightly et al. [39] found a positive relationship of LLD ≤20 mm with radiographic knee and hip osteoarthritis.

Total hip replacement surgery [40] usually results in mild postoperative LLD that could lead the prosthesis to higher pressures and potentially reduce the success of the surgery.

Evaluating which limb is bearing the greater load is a crucial issue after surgery because of the potential for stress to the prosthesis. It could contribute to patient dissatisfaction even when intervention had an outstanding result.

Furthermore, our research showed that stance times increased on the long leg when LLD increased. These findings are consistent with other studies [21,41]. The changes produced by mild LLD may appear minimal when viewed in simple measurements. However, they could generate major effects when it comes to repetitive loading. According to the meta-analysis by Crawford et al. [42], patients with foot plantar pressure overload and extended contact times have a high risk of developing a foot ulcer. The early evaluation of LLD with a pressure platform system in patients with diabetes mellitus could prevent ulcerations.

It should be noted that, in this study, lifts were always located under the right shoe. Some studies found differences between the dominant and non-dominant leg [43], which would be clinically interesting to test and evaluate in future studies. Furthermore, time and pressure parameters were evaluated for the total plantar surface. Previous studies have found intrasession variability when using regional analysis [27,44]. Future research should consider the study of asymmetries on pressure dynamic patterns as the predictive condition of LLD.

## 5. Conclusions

Increasing leg length discrepancy causes a decrease in both mean and peak pressure on the longer limb, and consequently, an overload on the short side. Furthermore, an increasing LLD causes an increased stance time on the long leg. Contrary to other studies, our findings suggest that an LLD smaller than 20 mm should not be ignored.

## Figures and Tables

**Table 1:** Technical specifications of the pressure platform.

Specification	Description
Size (Length/Width/Height)	615 × 565 × 23 mm
Weight	3.15 kg
Sensor type	Calibrated resistive
Active surface	400 × 400 mm
Sensor size	10 × 10 mm
Sensor number	1600 (40 × 40)
Minimum/maximum pressure range per sensor	0.4 N/m^2^ (0.0004 kPa) to 100 N/m^2^ (0.1 kPa)
Acceptable temperature	0 °C to +40 °C
Acquisition frequency	100 Hz
Power	Via Universal Serial Bus (USB) 5V, 400 mA
Operating system required	Windows^®^ 7, 8 or 10

**Table 2:** Descriptive data of the study participants showing demographics and anthropometric characteristics for the total sample by gender.

Variable	Male *n* = 13	Female *n* = 24	Total *n* = 37
	Mean ± SD (95% CI)	Mean ± SD (95% CI)	Mean ± SD (95% CI)
Age (years)	39.21 ± 11.52 (32.56–45.87)	38.91 ± 11.80 (33.81–44.02)	39.03 ± 11.54 (35.18–42.87)
Weight (kg)	73.50 ± 8.23 (68.74–78.25)	61.08 ± 10.13 (56.70–65.47)	65.78 ± 11.16 (62.06–69.50)
Height (m)	1.75 ± 0.09 (1.69–1.80)	1.64 ± 0.08 (1.61–1.68)	1.68 ± 0.10 (1.65–1.72)
MBI	23.99 ± 2.75 (22.40–25.58)	22.40 ± 2.83 (21.17–23.63)	23 ± 2.87 (22.04–23,96)
Foot size (EC)	42.03 ± 2.07 (40.83–43.23)	37.86 ± 1.20 (37.34–38.39)	39.44 ± 2.57 (38.58–40.30)

BMI, body mass index; SD, standard deviation; 95% CI, 95 percent confidence interval; EC, European countries.

**Table 3:** Intersession reliability of time and pressure variables for each foot under simulated LLD conditions.

Variable	Mean (SD)	CoV (%)	ICC (95% CI)	SEM%	MDC%
**0 mm of LLD**					
Stance time short (ms)	761.42 (7.19)	0.94	0.957 (0.932–0.976)	0.20	0.54
Stance time long (ms)	754.05 (2.96)	0.39	0.960 (0.936–0.977)	0.08	0.22
Mean pressure short (g/cm^2^)	748.74 (5.12)	0.68	0.955 (0.927–0.974)	0.15	0.40
Mean pressure long (g/cm^2^)	750.95 (12.37)	1.65	0.931 (0.889–0.960)	0.43	1.20
Peak pressure short (g/cm^2^)	1427.82 (16.46)	1.15	0.928 (0.886–0.959)	0.31	0.86
Peak pressure long (g/cm^2^)	1417.26 (13.03)	0.92	0.918 (0.869–0.953)	0.26	0.73
**5 mm of LLD**					
Stance time short (ms)	762.16 (5.77)	0.76	0.981 (0.969–0.989)	0.10	0.29
Stance time long (ms)	759.73 (5.03)	0.66	0.980 (0.968–0.988)	0.09	0.26
Mean pressure short (g/cm^2^)	746.61 (5.44)	0.73	0.955 (0.928–0.974)	0.15	0.43
Mean pressure long (g/cm^2^)	747.13 (11.88)	1.59	0.866 (0.785–0.923)	0.58	1.61
Peak pressure short (g/cm^2^)	1443.52 (14.39)	1.00	0.925 (0.879–0.957)	0.27	0.76
Peak pressure long (g/cm^2^)	1338.84 (9.45)	0.71	0.927 (0.883–0.958)	0.19	0.53
**10 mm of LLD**					
Stance time short (ms)	763.82 (14.15)	0.54	0.986 (0.977–0.992)	0.06	0.18
Stance time long (ms)	765.09 (4.23)	0.55	0.984 (0.974–0.991)	0.07	0.19
Mean pressure short (g/cm^2^)	756.19 (7.65)	1.01	0.970 (0.952–0.983)	0.18	0.49
Mean pressure long (g/cm^2^)	723.24 (11.83)	1.64	0.918 (0.869–0.953)	0.47	1.30
Peak pressure short (g/cm^2^)	1435.58 (11.95)	0.83	0.924 (0.878–0.956)	0.23	0.64
Peak pressure long (g/cm^2^)	1274.77 (15.55)	1.22	0.898 (0.837–0.941)	0.39	1.08
**15 mm of LLD**					
Stance time short (ms)	760.54 (4.39)	0.58	0.988 (0.981–0.993)	0.06	0.18
Stance time long (ms)	775.58 (4.19)	0.54	0.986 (0.977–0.992)	0.06	0.18
Mean pressure short (g/cm^2^)	760.41 (5.54)	0.73	0.972 (0.955–0.984)	0.12	0.34
Mean pressure long (g/cm^2^)	711.58 (5.25)	0.74	0.929 (0.886–0.959)	0.20	0.55
Peak pressure short (g/cm^2^)	1423.44 (17.28)	1.21	0.928 (0.884–0.958)	0.33	0.90
Peak pressure long (g/cm^2^)	1265.21 (17.58)	1.39	0.935 (0.895–0.962)	0.35	0.98
**20 mm of LLD**					
Stance time short (ms)	766.98 (5.22)	0.68	0.983 (0.976–0.990)	0.09	0.25
Stance time long (ms)	783.51 (4.31)	0.55	0.984 (0.974–0.991)	0.07	0.19
Mean pressure short (g/cm^2^)	758 (3.51)	0.46	0.962 (0.940–0.978)	0.09	0.25
Mean pressure long (g/cm^2^)	706.71 (5.47)	0.77	0.890 (0.824–0.937)	0.26	0.71
Peak pressure short (g/cm^2^)	1409.33 (21.37)	1.52	0.906 (0.850–0.946)	0.46	1.29
Peak pressure long (g/cm^2^)	1245.60 (16.16)	1.30	0.900 (0.841–0.943)	0.41	1.14

SD, standard deviation; CoV, coefficient of variation; ICC, intraclass correlation coefficient; 95% CI, 95 percent confidence interval; SEM, standard error of measurement; MDC, minimum detectable change.

**Table 4:** Repeated-measures analysis of variance (RM-ANOVA) including a test of influence of the sphericity assumption, analysis of effect, and a contrast analysis of the significant interaction effect.

Variable	MT *(p)*	SS	DF	MS	*F*	*p*	η^2^_p_
Stance time short ^a^	<0.001	682.97	2.18	313.00	0.30	0.758	0.008 †
Stance time long ^a^	<0.001	21043.48	2.09	10025.73	11.90	<0.001 **	0.249 †††
Mean pressure short	0.165 *	5330.90	4	1332.72	3.92	0.005 **	0.098 ††
Mean pressure long ^a^	<0.001	70469.07	2.63	26732.11	15.31	<0.001 **	0.299 †††
Peak pressure short ^a^	0.003	35130.29	2.79	12554.20	3.16	0.031**	0.081 ††
Peak pressure long ^a^	<0.001	754051.37	2.15	350219.50	32.54	<0.001 **	0.475 †††

MT(*p*), Mauchly test probability; SS, sum of squares; DF, degrees of freedom; MS, mean square; F, variance ratio; *p*, probability; η^2^_p_, partial eta squared; ^a^, Greenhouse–Geisser adjusted; *, sphericity assumed; **, reached level of significance; †, small effect; ††, medium effect; †††, large effect.

**Table 5:** Pairwise comparison based on estimated marginal means.

		STS		STL		MPS		MPL		PPS		PPL	
LLD (mm)		MD (SE)	*p* ^a^	MD (SE)	*p* ^a^	MD (SE)	*p* ^a^	MD (SE)	*p* ^a^	MD (SE)	*p* ^a^	MD (SE)	*p* ^a^
**0**	5	−0.73 (5.08)	1.000	−5.67 (4.40)	1.000	2.13 (3.87)	1.000	3.824 (8.10)	1.000	−14.80 (10.12)	1.000	78.41 (12.38)	<0.0001*
	10	−2.40 (7.28)	1.000	−11.03 (6.48)	0.973	−7.63 (4.89)	1.000	27.71 (9.48)	0.060	−6.86 (10.84)	1.000	142.49 (18.32)	<0.0001*
	15	0.88 (6.97)	1.000	−21.53 (6.78)	0.031 *	−11.66 (4.57)	0.15	39.37 (10.12)	0.004 *	3.47 (13.35)	1.000	152.05 (21.64)	<0.0001 *
	20	−5.28 (7.49)	1.000	−29.45 (6.68)	0.001 *	−9.25 (4.87)	0.66	44.24 (10.16)	0.001 *	16.68 (15.49)	1.000	171.65 (23.79)	<0.0001 *
**5**	0	0.73 (5.08)	1.000	5,67 (4.40)	1.000	−2.13 (3.87)	1.000	−3.82 (8.10)	1.000	14.80 (10.12)	1.000	−78.41 (12.38)	<0.0001 *
	10	−1.66 (3.89)	1.000	−5.36 (3.65)	1.000	−9.76 (4.50)	0.37	23.89 (6.95)	0.015 *	7.94 (10.64)	1.000	64.07 (14.81)	0.001 *
	15	1.62 (4.30)	1.000	−15.85 (4.16)	0.005 *	−13.79 (4.33)	0.03 *	35.55 (6.89)	<0.0001 *	18.27 (14.04)	1.000	73.63 (20.84)	0.011*
	20	−4.55 (4.91)	1.000	−23.78 (4.26)	0.0001 *	−11.38 (4.77)	0.23	40.42 (7.86)	<0.0001 *	31.48 (13.89)	0.3	93.24 (21.03)	0.001 *
**10**	0	2.40 (7.28)	1.000	11.03 (6.48)	0.973	7.63 (4.89)	1.000	−27.71 (9.48)	0.060	6.86 (10.84)	1.000	−142.49 (18.32)	<0.0001 *
	5	1.66 (3.89)	1.000	5.36 (3.65)	1.000	9.76 (4.50)	0.37	−23.89 (6.95)	0.015 *	−7.94 (10.64)	1.000	−64.07 (14.81)	0.001 *
	15	3.28 (3.63)	1.000	−10.49 (3.78)	0.087	−4.03 (3.75)	1.000	11.66 (5.77)	0.511	10.33 (10.15)	1.000	9.55 (12.77)	1.000
	20	−2.88 (3.70)	1.000	−18.42 (3.96)	0.0001 *	−1.62 (3.71)	1.000	16.53 (5.92)	0.083	23.54 (11.04)	0.4	29.16 (12.56)	0.261
**15**	0	−0.88 (6.97)	1.000	21.53 (6.78)	0.031 *	11.66 (4.57)	0.15	−39.37 (10.12)	0.004 *	−3.47 (13.35)	1.000	−152.05 (21.64)	<0.0001 *
	5	−1.62 (4.30)	1.000	15.85 (4.16)	0.005 *	13.79 (4.33)	0.03 *	−35.55 (6.89)	<0.0001 *	−18.27 (14.04)	1.000	−73.63 (20.84)	0.011 *
	10	−3.28 (3.63)	1.000	10.49 (3.78)	0.087	4.03 (3.75)	1.000	−11.66 (5.77)	0.511	−10.33 (10.15)	1.000	−9.55 (12.77)	1.000
	20	−6.17 (3.21)	0.63	−7.92 (2.76)	0.069	2.41 (3.41)	1.000	4.86 (6.46)	1.000	13.20 (13.20)	1.000	19.60(12.94)	1.000
**20**	0	5.28 (7.49)	1.000	29.45 (6.68)	0.001 *	9.25 (4.87)	0.66	−44.24 (10.16)	0.001 *	−16.68 (15.49)	1.000	−171.65 (23.79)	<0.0001 *
	5	4.55 (4.91)	1.000	23.78 (4.26)	0.0001 *	11.38 (4.77)	0.23	−40.42 (7.86)	<0.0001 *	−31.48 (13.89)	0.296	−93.24 (21.03)	0.001 *
	10	2.88 (3.70)	1.000	18.42 (3.96)	0.0001 *	1.62 (3.71)	1.000	−16.53 (5.92)	0.083	−23.54 (11.04)	0.4	−29.16 (12.56)	0.261
	15	6.17 (3.21)	0.63	7.92 (2.76)	0.069	−2.41 (3.41)	1.000	−4.86 (6.46)	1.000	−13.20 (9.66)	1.000	−19.60 (12.94)	1.000

STS, stance time short; STL, stance time long; MPS, mean pressure short; MPL, mean pressure long; PPS, peak pressure short; PPL, peak pressure long; LLD, leg length discrepancy; MD, mean difference; SE, standard error; *p*, Probability; ^a^, Bonferroni adjustment for multiple comparisons; *, reach level of significance.

## Data Availability

Not applicable.
